# Adolescent with Stroke-like Symptoms

**DOI:** 10.5811/cpcem.2018.2.36886

**Published:** 2018-04-05

**Authors:** Joseph C. Mazzei, Stephanie Louka, Ravindra Gopaul, Lindsay Taylor

**Affiliations:** Virginia Commonwealth University, Department of Emergency Medicine, Richmond, Virginia

## CASE PRESENTATION

**Image 1:**
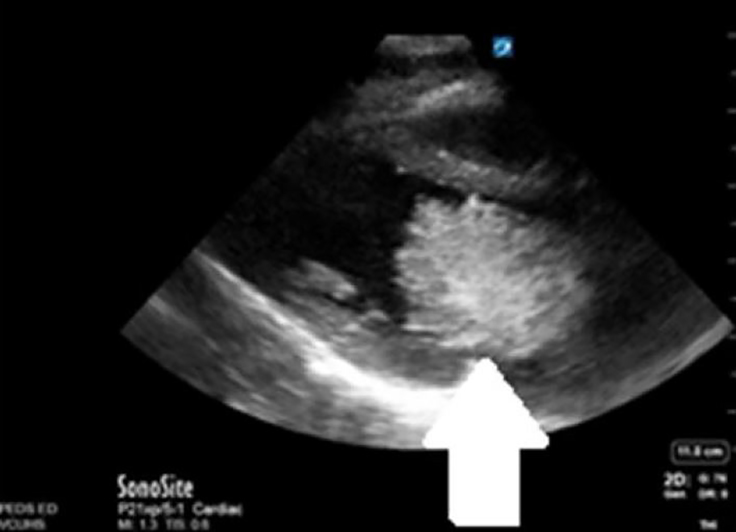
Parasternal long-axis view of left atrial myxoma (arrow).

**Image 2:**
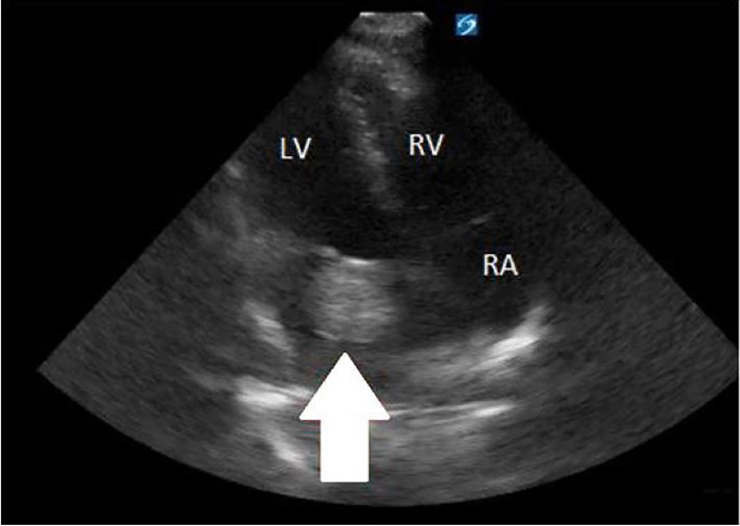
Apical four-chamber view of left atrial myxoma (arrow).

A 16-year-old male presented to the emergency department (ED) with left-sided weakness. He described having a headache with dizziness for seven days prior to presenting with new onset weakness. The patient was from Central America with no known medical history and was unvaccinated. The examination was significant for flaccid paralysis with decreased sensation in the left upper and lower extremities, plus a left-sided facial droop. The cardiac examination revealed a 2/6 diastolic murmur, loudest at the left sternal border. A point-of-care ultrasound performed in the ED demonstrated a left atrial mass swinging into the left ventricle during diastole (Images 1 and 2).

## DISCUSSION

**Image 3:**
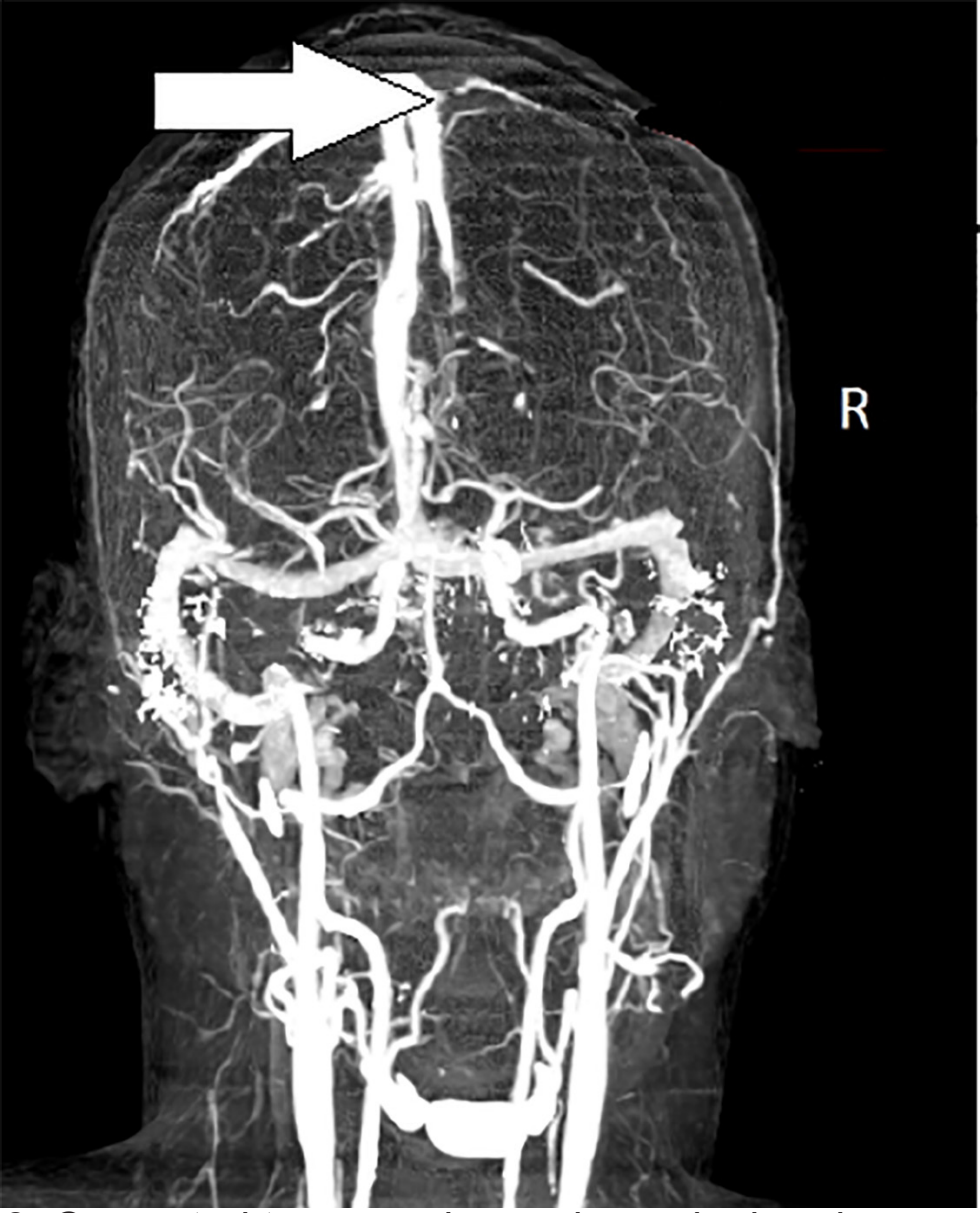
Computed tomography angiography head, coronal view demonstrating right middle cerebral artery (R-MCA) filling defect (arrow).

Computed tomography angiography of the head led to a diagnosis of left atrial myxoma with embolism resulting in non-hemorrhagic occlusion of the right middle cerebral artery (Image 3). After initial stabilization, the patient was taken to surgery for removal of a 52mm x 42mm benign, left atrial myxoma. Two months after presentation the patient had regained approximately 70% of his strength and full sensation.

Cardiac tumors are a rare diagnosis, with a reported incidence of 0.01–0.02%.^1^ The surface of these tumors is friable, which can lead to embolization in one-third of patients, resulting in devastating neurovascular injury and often sudden death.^2^ Intracardiac tumor diagnosis with transthoracic echocardiogram has a 95.2% detection rate.^3^ Given the high risk of morbidity and mortality, expeditious diagnosis and surgical resection is considered standard of care. Point-of-care ultrasound, especially with a critically ill patient like ours, has proven to be an invaluable tool in improving both immediate patient care and long-term prognosis.

**CPC-EM Capsule**

What do we already know about this clinical entity?

Cardiac myxoma is a rare, benign tumor of the heart that predisposes patients to embolic phenomena involving cerebral or mesenteric arteries typically requiring surgical repair.

What is the major impact of the images?

Point-of-care ultrasound (POCUS) visualization of a left atrial myxoma, and subsequent computed tomography of a cerebral artery-filling defect, solidified our diagnosis of tissue embolus, which explained the patient’s constellation of symptoms and led to timely surgical consultation

How might this improve emergency medicine practice?

This case highlights the utility of POCUS in the emergency department to quickly identify life-threatening ailments that may otherwise go undiagnosed.

Documented patient informed consent and/or Institutional Review Board approval has been obtained and filed for publication of this case report.
